# The Impact of Artificial Intelligence on Health Equity in Oncology: Scoping Review

**DOI:** 10.2196/39748

**Published:** 2022-11-01

**Authors:** Paul Istasy, Wen Shen Lee, Alla Iansavichene, Ross Upshur, Bishal Gyawali, Jacquelyn Burkell, Bekim Sadikovic, Alejandro Lazo-Langner, Benjamin Chin-Yee

**Affiliations:** 1 Schulich School of Medicine and Dentistry Western University London, ON Canada; 2 Rotman Institute of Philosophy Western University London, ON Canada; 3 Department of Pathology & Laboratory Medicine Schulich School of Medicine Western University London, ON Canada; 4 Library Services London Health Sciences Centre London, ON Canada; 5 Division of Clinical Public Health Dalla Lana School of Public Health University of Toronto Toronto, ON Canada; 6 Bridgepoint Collaboratory for Research and Innovation Lunenfeld Tanenbaum Research Institute Sinai Health System Toronto, ON Canada; 7 Division of Cancer Care and Epidemiology Department of Oncology Queen's University Kingston, ON Canada; 8 Division of Cancer Care and Epidemiology Department of Public Health Sciences Queen's University Kingston, ON Canada; 9 Faculty of Information and Media Studies Western University London, ON Canada; 10 Division of Hematology Schulich School of Medicine and Dentistry Western University London, ON Canada; 11 Division of Hematology Department of Medicine London Health Sciences Centre London, ON Canada

**Keywords:** artificial intelligence, eHealth, digital health, machine learning, oncology, cancer, health equity, health disparity, bias, global health, public health, cancer epidemiology, epidemiology, scoping, review, mobile phone

## Abstract

**Background:**

The field of oncology is at the forefront of advances in artificial intelligence (AI) in health care, providing an opportunity to examine the early integration of these technologies in clinical research and patient care. Hope that AI will revolutionize health care delivery and improve clinical outcomes has been accompanied by concerns about the impact of these technologies on health equity.

**Objective:**

We aimed to conduct a scoping review of the literature to address the question, “What are the current and potential impacts of AI technologies on health equity in oncology?”

**Methods:**

Following PRISMA-ScR (Preferred Reporting Items for Systematic Reviews and Meta-Analyses extension for Scoping Reviews) guidelines for scoping reviews, we systematically searched MEDLINE and Embase electronic databases from January 2000 to August 2021 for records engaging with key concepts of AI, health equity, and oncology. We included all English-language articles that engaged with the 3 key concepts. Articles were analyzed qualitatively for themes pertaining to the influence of AI on health equity in oncology.

**Results:**

Of the 14,011 records, 133 (0.95%) identified from our review were included. We identified 3 general themes in the literature: the use of AI to reduce health care disparities (58/133, 43.6%), concerns surrounding AI technologies and bias (16/133, 12.1%), and the use of AI to examine biological and social determinants of health (55/133, 41.4%). A total of 3% (4/133) of articles focused on many of these themes.

**Conclusions:**

Our scoping review revealed 3 main themes on the impact of AI on health equity in oncology, which relate to AI’s ability to help address health disparities, its potential to mitigate or exacerbate bias, and its capability to help elucidate determinants of health. Gaps in the literature included a lack of discussion of ethical challenges with the application of AI technologies in low- and middle-income countries, lack of discussion of problems of bias in AI algorithms, and a lack of justification for the use of AI technologies over traditional statistical methods to address specific research questions in oncology. Our review highlights a need to address these gaps to ensure a more equitable integration of AI in cancer research and clinical practice. The limitations of our study include its exploratory nature, its focus on oncology as opposed to all health care sectors, and its analysis of solely English-language articles.

## Introduction

### Background

Artificial intelligence (AI), a field that aims to create computers that can achieve human-like understanding and perform tasks normally associated with human intelligence, is finding increasing applications in health care and public health [1,2]. Machine learning (ML) is a form of AI that involves algorithms that draw on big data—data sets whose size go beyond the capabilities of standard data analysis software—to *learn* to make predictions [3]. Oncology has been the focus of significant AI research and development and serves as an important area to observe and assess the early integration of AI in health care [4]. AI applications in oncology are expanding to cover a wide range of uses, from pathology and diagnostic imaging to clinical risk prediction and treatment planning for several types of cancer [5-7].

Despite its promise, the use of AI in health care raises several ethical issues, most notably concerns over bias and the potential for AI systems to adversely impact health equity. Health equity has been defined as “the absence of systematic disparities in health between groups with different levels of underlying social advantage/disadvantage” [8]. Studies have demonstrated how the use of biased data sets in training ML algorithms can exacerbate health inequities [9-11]. For example, Obermeyer et al [11] revealed how an ML algorithm trained to predict health risk consistently underestimated the health of Black patients because of the use of health care cost as a proxy for health. However, others have argued that AI systems can help illuminate health inequities and, if used correctly, may help address existing disparities [12-15]; for example, AI has been used to analyze search engine results from 54 African nations to guide resource allocation and improve access to care [15]. It is no wonder that a recent report from the Wellcome Trust on the ethical, social, and political challenges of AI in health care was not able to reach a clear consensus on the impact of AI on health equity [16]. Moreover, despite cancer being a major focus of AI research and development, the impact of AI on health equity in oncology remains underexplored. There is growing literature characterizing the problems of health disparities in oncology, which range from issues of access to high-quality care and research to structural barriers in health promotion and the lack of awareness of existing health inequities [17]. Given the expanding use of AI in oncology, there is an urgent need to assess the interplay between AI technologies and health equity in oncology to better understand the social and ethical dimensions surrounding the integration of AI.

### Objective

This scoping review of the literature aimed to address the question, “What are the current and potential impacts of AI applications on health equity in oncology?” We analyzed the literature on contemporary AI applications in oncology with a focus on implications for health equity to identify recurring themes as well as important gaps and areas for future research.

## Methods

### Overview

Our scoping review protocol followed previously established methods [18] with reporting in accordance with the PRISMA-ScR (Preferred Reporting Items for Systematic Reviews and Meta-Analyses extension for Scoping Reviews) framework [19].

### Search Strategy

We used a sensitive search strategy to identify a representative sample of the available literature on the influence of AI on health equity in oncology. On the basis of a combination of synonymous searches comprising controlled vocabularies, such as Medical Subject Headings in MEDLINE or EMTree descriptors in Embase, and free-text terms using alternative word spellings and endings for the 3 core concepts: AI (*algorithm, machine learning, artificial intelligence, deep learning, and convolutional neural networks*), equity (*health equality, health inequality, health disparity, and socioeconomic factors*), and oncology (*neoplasm, cancer, squamous, and metaplasia*), which informed a comprehensive search strategy developed by the clinical librarian (AI) with experience in conducting electronic literature searches on the recommendations from the review authors (PI, ALL, and BCY). We searched both databases (MEDLINE and Embase, via the OVID platform) from January 2000 to August 2021, and a preliminary search was performed on December 4, 2020. A detailed description of our search strategy is provided Multimedia Appendix 1.

### Eligibility Criteria and Article Screening

In addition, the web-based search engine Google Scholar was used to identify additional potentially relevant studies that were not indexed in bibliographic databases. The bibliographies of all relevant retrieved articles were also examined to identify further relevant studies. To capture the breadth of literature on AI and health equity in oncology, we did not impose limits based on study type and included clinical studies—that is, studies in which AI was applied and evaluated for a specific clinical intervention, whether it be diagnostic, prognostic, screening, or treatment planning—commentaries and opinion articles. Limits were imposed for English-language-only articles, as it was the main language of proficiency for the research team, thus allowing for detailed and critical examination of the selected articles to take place. All identified records from the electronic search were imported into Covidence systematic review software (Veritas Health Innovation) for further analysis and screening.

After duplicate records were removed, 2 reviewers (PI and WSL) independently screened the titles and abstracts of selected records using the inclusion and exclusion criteria, which were defined a priori: records were selected during the title and abstract screening if they mentioned the core concepts (AI, health equity, and oncology) or related terms. Abstracts were excluded if they did not meet the inclusion criteria or if they involved nonhuman participants. All conflicts were resolved by a third reviewer (BCY). The list of selected abstracts was then reassessed by all 3 reviewers (PI, WSL, and BCY) in full-text reviews to identify records related to the research question. Records that generated a unanimous consensus were selected for full-text review, whereas those that did not engage with the 3 key concepts were excluded. Conflicts were resolved through discussion between all 3 reviewers. A further full-text review was conducted by all 3 authors, further applying the eligibility criteria.

### Data Extraction and Analysis

Data extraction and analysis involved both descriptive and qualitative components. Descriptively, we extracted data on the year of publication, country of affiliation of the senior author, type of institution of affiliation of the senior author, type of study, type of AI, cancer type, and, when available, the cost of the proposed technology. The country of affiliation of the senior author was classified as high income, low income, and middle income following the most recent United Nations classification [20]. Qualitatively, we analyzed articles for emerging themes related to health equity in oncology, inherent assumptions, and gaps in the literature. Thematic analysis followed the steps outlined by Braun and Clarke, which have been widely applied in scoping reviews of qualitative research, including in health care [21], to generate a comprehensive thematic representation of a given area of research [22]. This process involved familiarization with the data set of included articles, generation of initial codes, collation of codes into provisional themes, review of themes in relation to initial codes, and the entire data set, followed by definition and naming of each theme to generate a comprehensive representation of the data. Steps of data familiarization and initial coding were performed independently by 3 reviewers (PI, WSL, and BCY); steps of collation, review of themes, and definition and naming were performed through discussion between study coauthors. Articles that had insufficient engagement with 3 key concepts, that is, those that mentioned the issues of bias or equity but did not elaborate on specific issues arising from AI, or made insufficient links between the core concepts, that is, those that mentioned all 3 core concepts but had no further exploration of their relationships, were excluded.

## Results

### Selection and Characteristics of Sources of Evidence

**Figure 1:**
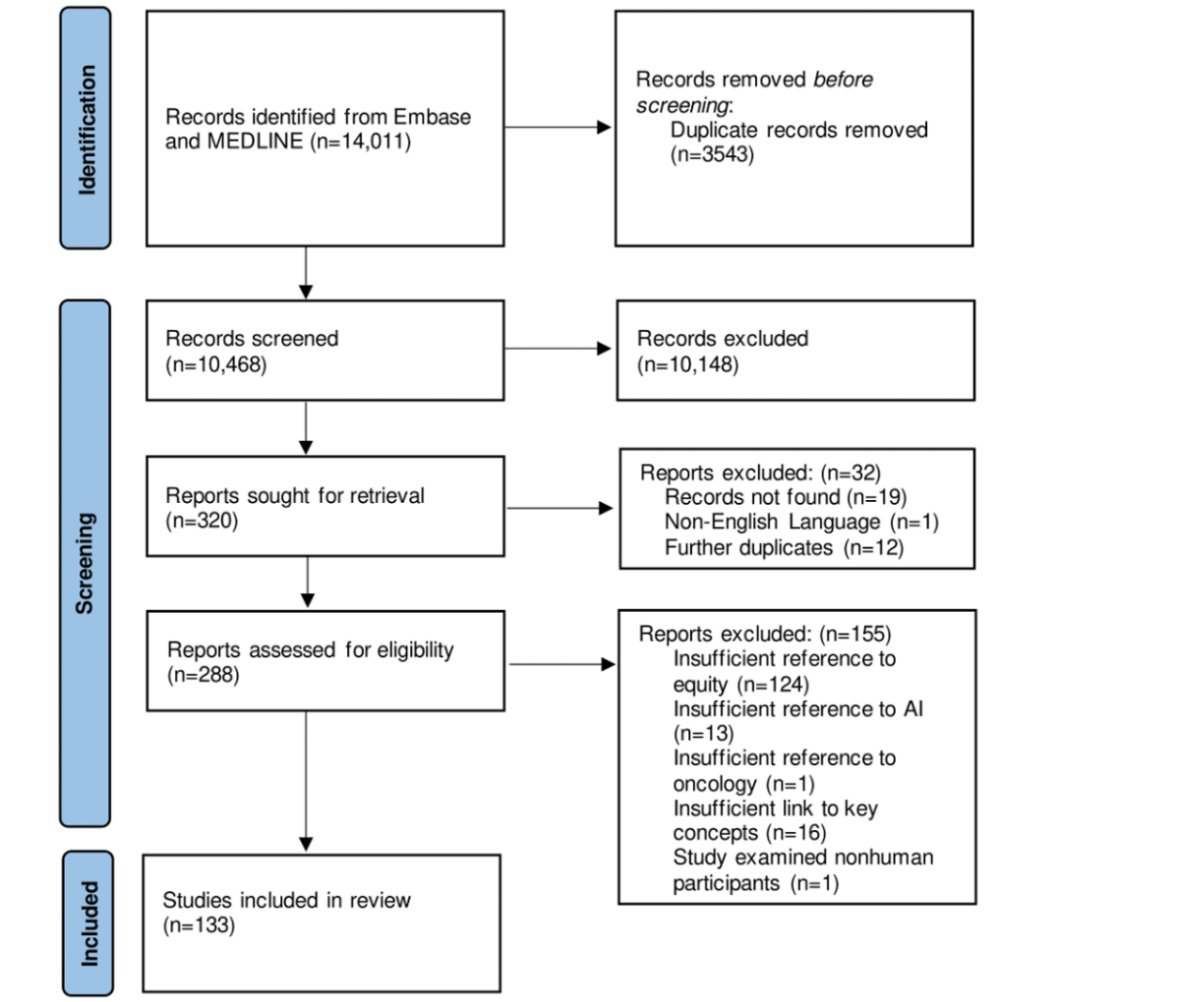
PRISMA-ScR (Preferred Reporting Items for Systematic Reviews and Meta-Analyses extension for Scoping Reviews) flow diagram for the identification of studies via databases and registers. AI: artificial intelligence.

**Figure 2:**
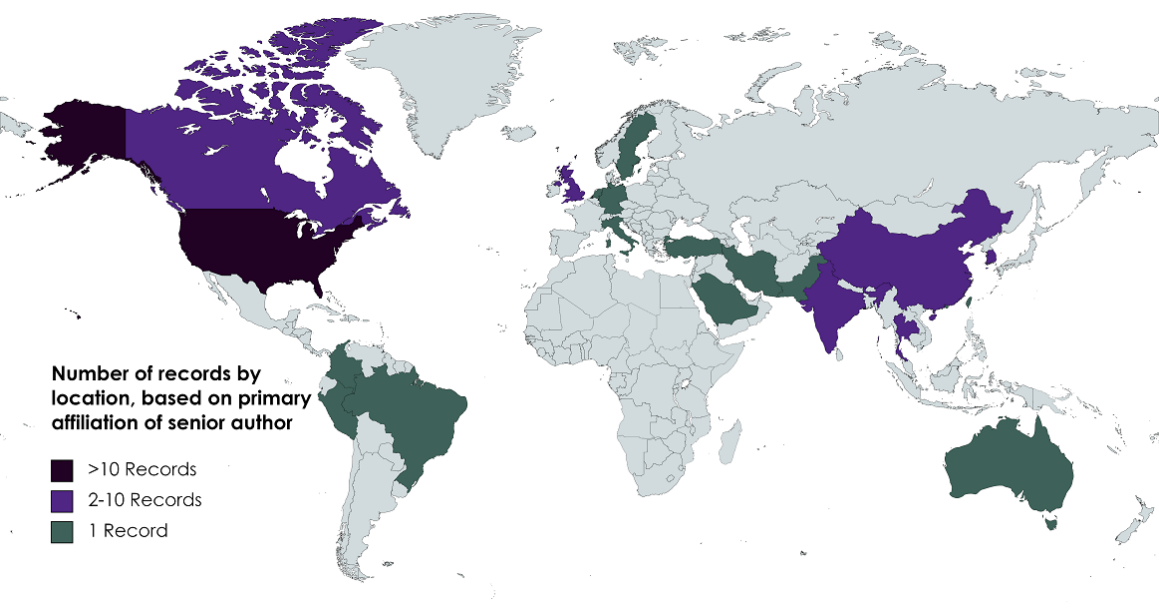
Country of affiliation of senior author (map created with MapChart).

Our search yielded 14,011 records. After removing duplicates, 10,468 records were screened, and 133 articles met the inclusion criteria [4,23-154] (Figure 1). All the records included in our review were published between 2010 and 2021, with the majority (124/133, 93.2%) published after 2018 (Table 1). Although a range of countries, based on the affiliation of the senior author, were represented in our review (Figure 2), most were from the United States (90/133, 67.7%). The majority were from academic centers (121/133, 90.9%), with a minority from the government, nonprofit organizations, and industry (Table 1). Approximately half of the records involved clinical studies (68/133, 51.1%), whereas the rest were epidemiological studies, commentaries, surveys, and interviews. Most of the records drew on ML techniques to address their research question: 12.8% (17/133) records discussed AI in general; 30.8% (41/133) records did not specify the type of ML used or used multiple ML algorithms; 47.4% (63/133) used supervised ML algorithms; and a smaller subset (4/133, 3%; 6/133, 4.5%; and 2/133, 1.5%) used unsupervised ML, natural language processing, and reinforcement ML, respectively. AI was used for a wide range of applications and often a combination of applications, including epidemiological (28/133, 21.1%), diagnostic (25/133, 18.8%), prognostic (25/133, 18.8%), and screening (25/133, 18.8%; Table 1).

**Table 1:** Characteristics of studies included in the scoping review (n=133).

Study characteristics			Studies, n (%)
**Year of publication^a^**			
	2000-2017	9 (7.5)	
	2018	13 (9.8)	
	2019	21 (15.8)	
	2020	48 (36.1)	
	2021	42 (31.6)	
**Type of study**			
	Clinical	62 (46.6)	
	Epidemiological	40 (30)	
	Review	15 (11.3)	
	Commentary	11 (8.3)	
	Survey and interviews	5 (3.8)	
**Institution type^b^**			
	Academic	121 (91)	
	Governmental and nongovernmental organizations	12 (9)	
**Type of artificial intelligence application^c^**			
	Screening	41 (30.8)	
	Diagnostic	41 (30.8)	
	Therapeutic	15 (11.3)	
	Prognostic	45 (33.8)	
	Epidemiological	45 (33.8)	
**Type of cancer**			
	General	28 (21.1)	
	Gynecologic	19 (14.3)	
	Breast	16 (12)	
	Oral	12 (9)	
	Prostate	12 (9)	
	Skin	12 (9)	
	Lung	8 (6)	
	Hematologic	6 (4.5)	
	Brain	4 (3)	
	Liver	4 (3)	
	Colorectal	3 (2.3)	
	Esophageal	3 (2.3)	
	Head and neck	2 (1.5)	
	Pancreatic	2 (1.5)	
	Gastrointestinal	1 (0.8)	
	Thyroid	1 (0.8)	

^a^Inclusive.

^b^On the basis of affiliation of the senior author.

^c^Total numbers exceed 133 due to 26 articles falling into multiple categories.

### AI Applications in Specific Cancer Types

Studies from our review investigated a wide range of cancers, with general oncological applications being the dominant category (28/133, 21.1%), followed by gynecologic (19/133, 14.3%), breast (16/133, 12%), oral (12/133, 9%), prostate (12/133, 9%), and dermatologic cancers (12/133, 9%). Among the articles on gynecologic cancers, 84% (16/19) were categorized under theme 1, discussing the use of AI technologies to address disparities in gynecologic cancer screening (11/16, 70%) [23,84-93], diagnosis (4/16, 25%) [94-97], and treatment (1/16, 6%) [98]. Of the 16 articles, 15 (94%) developed AI technologies to target gynecologic cancer disparities in low- and middle-income countries (LMICs) [84-98], while 1 (6%) did so for implementation in high-income countries (HICs) [23]. The other 3 (n=19, 16%) articles fell under theme 3, discussing the use of AI to explore the genetic (1/3, 33%) [99] and social (2/3, 67%) determinants of health outcomes in gynecologic cancers [100,101]. Moreover, most of these articles were clinical studies (14/19, 74%) [84-89,93-95,97-101], 16% (3/19) were commentaries [23,90,91], 5% (1/19) was an epidemiological study [96], and 5% (1/19) was a review [92].

Articles examining breast cancer have discussed a broader range of themes relating to health equity. Of the 16 articles, 6 (38%) focused on theme 1 [24,102-106], with all 6 looking at the applications of AI in LMICs. Of the 16 articles, 2 (13%) fell under theme 2: one discussed the use of AI to mitigate bias [107], whereas the other raised the issue of how AI might exacerbate and mitigate biases in breast cancer diagnoses [108]. Of the 16 articles, 7 (44%) fell under theme 3, with 6 (86%) examining the link between social determinants [109-114] and 1 (14%) examining the link between genetic determinants of health and breast cancer [115]. Of the 16 articles, 1 (6%) fell under multiple themes [116]. In addition to touching on a wider variety of themes than gynecologic cancers, articles examining breast cancer were also more varied: 44% (7/16) were clinical studies [24,102,103,110,112-114], 25% (4/16) were epidemiological studies [104,109,111,115], 25% (4/16) were reviews [106-108,116], and 6% (1/16) was a commentary [105].

### Critical Appraisal Within Sources of Evidence

We identified three main themes related to the impact of AI on health equity in oncology: (1) the development of AI technologies to reduce health disparities faced by populations in both LMICs and HICs; (2) the concern that biased AI algorithms might exacerbate health inequities counterposed by the hope that AI technologies might help overcome human biases; and (3) the power of AI to uncover biological and social determinants of health in oncology. Themes were further broken down into subthemes, where applicable. A full list of the articles categorized by theme can be found in Multimedia Appendices 2-5.

### AI and Health Disparities

#### Overview

The most prominent theme in our analysis, based on the number of records, was the development of AI technologies to address health disparities in oncology (58/133, 43.6%). This included the use of AI to address disparities in access to screening, diagnostic, and therapeutic technologies for underserved populations in LMICs (53/133, 39.8%) and minority populations in HICs (3/133, 2.3%). Of 133 studies, 2 (1.5%) used AI to address disparities in both LMICs and HICs. A total of 16 articles on this theme were commentaries or reviews discussing multiple applications in cancer care. Of the 58 articles, 17 (29%) were described as pilot studies. We further divided this theme into several subthemes based on the type of AI technology, including AI applications, to analyze the genomic, histological, radiographic, image, and demographic data.

#### Using AI to Address Disparities in Cancer Screening and Diagnosis

The literature under this theme highlighted how technologies could improve the delivery of health care to disadvantaged populations in both LMICs and HICs. In LMICs, these technologies were aimed at rectifying 2 main problems: addressing health care personnel shortages, thereby reducing the bottleneck effect created by a low ratio of health care professionals to the populations they serve and overcoming constraints resulting from limited medical equipment [117]. For example, point-of-care and smartphone-based technologies for oral cancer screening in low-resource settings aim to address the bottleneck effect created by a low number of health care professionals [118]. One example of AI technology aimed at addressing constraints from limited medical equipment is a mobile-based oral cancer image analysis software for use in rural India [119]. In the absence of a stable internet connection, the AI algorithm can analyze images directly on a smartphone, which are then uploaded to a cloud server and assessed by a remote specialist when internet is available. AI applications to address health disparities in oncology in HICs was a less explored topic, with some articles discussing algorithms to selectively target disadvantaged populations [120,121]. For instance, given the high prevalence of oral cancer in South Asian populations [155], 1 study used ML to develop a quantitative cytology program to selectively improve oral cancer screening among South Asians living in British Columbia, Canada [120].

The development of AI aimed at reducing health disparities drew on a range of data, from genomics and imaging to demographic data, all aimed at reducing demands on underresourced health care systems and improving the available medical equipment. One example is an AI image analysis algorithm for breast cancer detection that improves screening in underserved and low-resource settings by applying deep learning to novel ultrasound techniques [105,106,114]. Finally, AI has also been applied to address disparities in access to diagnostic pathology; these included examples such as decision support systems to assist with histopathological diagnosis of brain tumors in resource-poor settings [122] and image analysis of cervical lesions [97].

Studies have reported a range of outcomes, with screening and diagnostic technologies showing a wide variation in sensitivity (75%-100%), specificity (71%-100%), and accuracy (61%-100%). Most studies on this topic (43/58, 74%) offered no comparison between the performance of the proposed technology and the existing standard of care. When the AI algorithms were directly compared with the standard of care, the results varied. Most of the articles noted no difference between AI algorithms and the standard of care [93,97,123-125], whereas others observed that the accuracy of AI algorithms was lower than that of human physicians [85,126]. One study noted that AI outperformed its human counterpart when detecting and staging prostate cancer in a higher number of patients [117].

#### Gaps and Challenges With Using AI to Address Health Disparities

Although several articles have highlighted how AI might help address health care shortages in LMICs, a recurrent problem noted in the literature is the lack of consideration for the infrastructure and human resources necessary to implement these AI technologies. To support the use of digital technologies, and specifically AI, LMICs require both health care providers trained to use specific technologies and sufficient technological infrastructure, including buildings where the hardware can be housed and cables to carry digital signals leading to widespread and stable internet access; in other words, the performance of AI algorithms is intertwined with sociotechnical factors [156,157]. Although HICs may have existing technological infrastructure to implement AI technologies more readily, LMICs often lack such infrastructure [158]. Considerations such as the cost of implementation and the need for maintenance and ongoing support once implemented, the need for trained personnel to use AI technologies, and the need for technological support to allow for the integration of the developed AI technologies were rarely discussed by articles in our review. Only select articles mentioned the lack of infrastructural considerations in the development of AI technologies [87,117,119,127,128]. For example, Anirvan et al [129] noted that, “while in developed countries with a well-equipped health care model in place this may not be a problem, in poor, rural, and resource-constrained settings, it may aggravate the burdened health care system in place.”

In addition, our review identified equity issues related to the cost of AI technologies; such technologies can be costly and may not be affordable in many LMICs under existing economic circumstances. Love et al [102] developed an AI device to triage breast lumps in low-resource settings but noted that “the device used in this study is more expensive than most LMICs settings can afford, lower cost devices are becoming more available.” However, others were able to create technologies that may be more affordable for LMICs: a gene expression assay costing US $450 capable of assessing samples for only US $10 [130].

To ensure that AI technologies designed for HICs can be effectively applied in LMICs, collaboration between these 2 settings is invaluable. Of the 42 studies that were conducted in LMICs, 11 (26%) were led by research groups from LMICs in question, and from the remaining 31 records, 27 involved collaboration with coauthors from the specific LMIC. When such a collaboration occurred, AI technologies were primarily designed in HICs and implemented in LMICs. This divide between the location of development and location of the implementation of AI in global oncology can pose a barrier to integration in LMICs due to costs [102] and infrastructural considerations [88], thereby suggesting a need for greater attention to co-design, which refers to the involvement of end users in the design process of AI technologies [159]. Moreover, it is important to recognize that the inclusion of researchers from LMICs in the design of AI technologies alone does not guarantee widespread improvements in health for patients in these countries. Rather, benefits are often limited to select partner sites of HICs; therefore, while these technologies may help address global disparities, they may exacerbate inequities within LMICs [130]. To ensure a more equitable distribution of benefits within LMICs, research should extend beyond specific partner institutions, engaging additional stakeholders from relevant government and nongovernmental organizations to evaluate and implement technologies. However, as noted in our review, there was only limited involvement of nonacademic institutions in the articles included in our review.

One additional problem that has been raised surrounding the use of AI in LMICs is the issue of data colonialism [160], a practice in which data are extracted from LMICs by institutions in HICs for the purposes of building algorithms whose benefits accrue primarily to stakeholders in HICs [161]. Although articles from our review did not engage directly with these issues, some did discuss important considerations for what collaboration means between HICs and LMICs [85,97,130,131]. However, there was limited acknowledgment of ethical issues arising from the involvement of LMICs as mere resources for data extraction and algorithmic training or as an exploratory ground for novel applications of AI technologies for global health.

### AI and Bias

#### Overview

The second theme identified in our review relates to the issue of bias. Bias in AI is a widely discussed topic and has the potential to exacerbate health disparities across different populations; while bias is an inherent feature of all AI systems, the main types of bias of ethical concern are those biases arising in algorithmic development or data sets [162] that can result in individuals being treated unfairly based on particular characteristics [163]. In a similar vein, 1 article in our review distinguished between concepts of desirable and undesirable biases, whereas desirable biases are those that take group data into consideration to account for base-rate differences and undesirable biases are those that are developed based on inaccurate or incomplete data, which in turn leads to group discrimination [132]. For instance, total melanoma rates are higher in men than in women [164]; thus, a desirable bias would include a training sample for an AI algorithm used to detect melanoma purposefully *biased* (desirably) to contain more men than women, representing the base rates of melanoma incidence. The authors suggest the use and integration of desirable biases to promote gender equity in health care while decreasing undesirable biases.

With rising concerns surrounding bias in AI [9-11], and conversely, the hope that AI algorithms may be able to help mitigate bias in human judgment [12,13,15], we expected to see a much larger number of articles discussing this issue; however, only 12% (16/133) articles directly engaged with the theme of bias. These articles fell into 2 main categories: those that explored how AI algorithms might help mitigate biased judgments in physicians’ clinical practice (5/133, 5%) and those that argued that AI trained on biased data sets can exacerbate existing inequities (10/133, 7.5%), while 1 article (1/133, 0.8%) focused on both subthemes.

#### The Use of AI to Uncover Bias in Clinical Practice

The use of AI technologies in health care can uncover biases in both data sets and physicians’ actions. For instance, head and neck cancers may develop spontaneously or in association with human papillomavirus (HPV), and characterization of such cancers as HPV-associated can affect treatment decisions [165]. Patients diagnosed with HPV-positive versus HPV-negative head and neck cancers have different demographic features, with younger individuals and individuals with more sexual partners being overrepresented in the HPV-positive group [166]. D’Souza et al [133] thus used AI to assess the use of clinical and demographic characteristics as diagnostic predictors of HPV-positive and HPV-negative head and neck cancers. However, these authors noted that clinical and demographic characteristics had only *moderate* accuracy in predicting HPV status, leading to a potential bias in treatment if these variables were used to predict HPV status without further investigation. In addition, AI can be used to uncover the biases found in data sets. Howard et al [134] deployed a deep learning model to assess institutional biases in data submitted to The Cancer Genome Atlas. They noted that biased digital histological signatures can stem from specific features of the institutions from which the data originate. AI algorithms may then provide prognostic information based on these institution-specific signatures rather than on the intrinsic histology of the sample.

#### The Use of AI to Mitigate Bias in Clinical Practice

We also identified articles that discussed the use of AI to mitigate bias in clinician decision-making. In criticizing the Fitzpatrick scale in dermatology, Okoji et al [135] argued that AI-based approaches might lead to a more objective classification system for skin typing. AI systems can identify subtle variations that are not visible to the human eye, thereby leading to more equitable dermatological assessments. However, a major caveat was the lack of discussion surrounding the populations used to train these AI algorithms in dermatology. For instance, several studies included predominantly White populations or did not specify the racial and ethnic makeup of the population used to develop their algorithms [124,136,167]. Only 1 article in our review specifically addressed this problem: to counterbalance the skewed nature of dermatologic data available for AI training, Pangti et al [137] sought selective patient populations to train an AI algorithm to detect skin diseases using locally generated data from India. As medical AI systems are prone to generating biased results that lead to disparities between ethnic groups, some authors proposed that stratification for minority communities that suffer from underrepresentation in training data sets could help rectify this bias [108]. Instead of a one-size-fits-all model, AI programs can be developed to target specific subpopulations. For instance, Gao and Cui [138] suggested the use of transfer learning, an AI training technique whereby knowledge gained from training an AI system on a larger data set, for example, a majority ethnic group, is transferred to be applied to a smaller data set, such as a minority ethnic group [138]. This technique attempts to compensate for missing data from “data-disadvantaged ethnic groups by leveraging knowledge learned from other groups with more abundant data” [138]. Yet, as the authors note, data inequality remains a central issue in training ML algorithms in multiethnic populations, and differential accuracy in performance between ethnic groups is an ongoing challenge.

#### Biased Data Sets and Biased AI

The final category in this theme was articles discussing the use of biased data sets to train AI algorithms; surprisingly, few articles discussed this topic. For instance, Khor et al [139] used a data set with racial demographics of 53% non-Hispanic White, 22% Hispanic, and 13% Black or African American to develop a recurrence risk prediction model for adults with prostate cancer. Even with the explicit inclusion of race, they noted that the model had “worse performance in minority subgroups compared to NHW [non-Hispanic White].” Conversely, others argued that bias in training data sets of AI algorithms may not always result in decreased generalizability; for example, Gilson et al [140] suggested that biased gender representation in training data sets did not lead to decreased generalizability in an algorithm to predict survival in non–small cell lung cancer.

#### Gaps in the Discussion of AI and Bias

Overall, engagement with issues of bias resulting from the use of AI in oncology was limited, an unexpected finding, given that this concern is widely discussed elsewhere in the literature on AI ethics and may act as a mechanism through which AI systems exacerbate health inequities. Our findings suggest that bias remains an underexplored topic in the literature on AI in oncology. It is also worth noting that the few articles that mentioned bias often did so briefly in their limitations section, usually in reference to how biased data sets might impact the validity and generalizability of AI algorithms but without further engagement with how these issues might be mitigated or addressed by future research.

### AI and Determinants of Health Outcomes

#### Overview

The final theme identified in our review was the use of AI to investigate the determinants of health outcomes in oncology. A total of 41.4% (55/133) articles fell under this theme and were divided into subthemes based on the determinants of health examined, ranging from biological variables (9/133, 6.8%) to social determinants of health (43/133, 32.3%), whereas 2.3% (3/133) articles focused on both themes. This category can be understood as the use of AI as an extension of traditional statistical models in clinical and epidemiological research in oncology.

#### AI and Biological Determinants of Health

Several articles under this theme applied AI to genomic data to predict outcomes in patients with cancer. For instance, Li et al [141] applied AI to genomic analysis across 3 racial groups to identify the impact of differential gene expression on racial disparities in cancer prevalence. They found differential gene expression in several cancers between racial groups, which they interpreted as supporting a genetic basis for racial differences in cancer prevalence.

#### AI and Social Determinants of Health

Although several studies have similarly applied AI in a reductionist manner, for example, to look for a genetic basis of health disparities [115,142], others have used AI to examine additional individual, environmental, and societal factors contributing to differential health outcomes between populations. Several articles in our review applied AI to shed light on the influence of race and socioeconomic status on health outcomes in oncology. For example, An et al [143] used an ML algorithm to examine the risk factors for the development of hepatocellular carcinoma in a Korean cohort, noting that higher income is associated with a lower risk of developing hepatocellular carcinoma. Bibault et al [144] applied AI to satellite imagery to investigate the relationship between socioeconomic status and cancer prevalence, observing that “satellite features are highly correlated with individual socioeconomic and health measures that are linked to cancer prevalence.” Several studies have suggested that applying AI to demographic data could help provide more comprehensive risk stratification models in oncology [112,168,169].

AI has also been used to identify racial disparities in cancer outcome. Tossas et al [101] used AI to predict populations at risk of delayed diagnosis of cervical cancer. They noted that more than half of the patients with a late cancer diagnosis were African American, findings that they argue can be used to target cervical cancer screening. Others have also used AI to examine outcomes following neurosurgery for brain tumors, noting that minority race is an independent risk factor for an extended length of stay and increased cost [145,146].

AI has also been applied to examine the influence of rural and urban residences on cancer prevalence and outcomes. Rural residences are known to influence access to cancer treatment, with novel therapies often concentrated in academic centers located in urban settings [170]. The impact of rural residence on cancer outcomes was investigated by Zhong et al [112], who used AI to create personalized prognostication models for early invasive breast cancer in a Chinese cohort. By incorporating residential status in their algorithm, the group found that despite lower rates of breast cancer in rural populations, the associated mortality risk was significantly higher. Aghdam et al [147] used the AI algorithm to study access to stereotactic body radiation therapy for prostate cancer and noted that travel distance did not prevent access to stereotactic body radiation therapy for rural patients, suggesting that income and race may be more important determinants of access to treatment.

#### Gaps in Using AI to Investigate Determinants of Health

For most studies in our review, there was a lack of justification for the use of AI and, more specifically, a lack of discussion as to why particular AI algorithms were chosen and their advantages over other statistical methods to address a given research question. AI algorithms are undeniably powerful tools for analyzing large amounts of data and selecting articles that mention the benefits of AI over other statistical methods [143,144,167,168]. However, others have argued that the use of AI has not yielded better risk prediction models compared with traditional statistical methods [169]. In their review on the efficacy of AI as opposed to traditional statistics in medicine, Rajula et al [145] noted that the latter seemed to be more useful when the number of participants significantly outweighed the number of variables in question, whereas the former is more suitable in fields with a large quantity of data, such as omics or radiodiagnostics. In light of this discussion, further justification for the use of AI to address specific research questions in oncology should be undertaken.

## Discussion

### Principal Findings

In this review, we evaluate the literature on the impact of AI on health equity in oncology. We identified 14,011 records in our search, of which 133 (0.95%) were substantially engaged with the core concepts of AI, health equity, and oncology. Our literature review revealed three main themes related to how AI technologies can (1) help address health disparities, (2) mitigate or exacerbate biased decision-making, and (3) elucidate the biological and social determinants of cancer outcomes. These themes relate to several issues discussed in the literature on AI and health equity in oncology and health care.

The first main theme noted in our review is how AI technologies can help address health disparities, both in LMICs and HICs. Previous scholarship examining the application of AI in global oncology has shed light on numerous practical and ethical challenges that have been discussed in the literature [171]. The existence of a “digital divide,” often cited as a key barrier to the implementation of AI technologies in global health, refers to the inequitable distribution of digital technologies, such as computational power, technical infrastructure, and data storage, that is required to use AI technologies [171]. Without prioritizing investment in the basic infrastructure, such as appropriate hardware to run AI programs, buildings where such hardware can be housed, and cables to carry digital signals, the utility of these technologies in the global health context should be questioned [172,173]. A number of articles identified in our review engaged with these voiced concerns, with some researchers creating technologies with the infrastructural capacities of specific LMICs in mind and others highlighting the need for additional infrastructure to support the technology they developed [87,102,129,130,148].

Another barrier to the implementation of AI technologies in LMICs discussed in the literature is the lack of generalizability of algorithms primarily designed in HICs but applied in LMICs [170]. As some researchers have observed, data used for training AI algorithms in HICs are “notorious for their lack of diversity, and concerns have been raised about their applicability even in their home countries” [172]. These data are often skewed toward the populations, diseases, and treatments available in countries training and developing AI technologies, thereby decreasing their generalizability to populations in LMICs. Articles from our review addressed this issue, voicing concerns about the applicability of AI algorithms developed in HICs to LMICs [108,110,134,143,149-151].

Our review also focused on solutions to the challenges posed by the integration of AI technologies in a global health context, which have been proposed elsewhere in the literature, with the predominant one being greater collaboration between HICs and LMICs in the development of AI technologies [171-173]. AI technologies created without appropriate consultation with the populations they are intended to serve may be highly inapplicable, impractical, and unethical. For example, treatment patterns produced by Watson for Oncology, an AI decision support system trained by data and experts from the Memorial Sloan Kettering Cancer Center, may be inapplicable to many LMICs [173]. In previous studies investigating this issue, some researchers have argued for the co-design of AI technologies, which requires the involvement of end users—and specifically marginalized groups—in AI research and development to ensure the equitable distribution of the benefits of these technologies [159,174].

To improve collaboration in global health research, others have proposed that journals publishing research conducted in LMICs have the responsibility of ensuring that at least one author involved in the study is from the countries in question [175]. We observed that this standard was met in most studies conducted in LMICs included in our review (27/31, 87%). However, further steps are required to ensure meaningful collaboration with investigators and stakeholders in LMICs, beyond simple inclusion in authorship, which risks fostering tokenism. As discussed earlier, this is especially important in AI research and development focused on addressing global health inequities in oncology, which needs to engage additional stakeholders beyond select partner sites to ensure fair distribution of benefits throughout populations [130,175]. This lacuna identified by our review reflects a broader lack of global coordination in AI research to set priorities and ensure fair distribution of research opportunities and resources, which is essential to prevent AI research from perpetuating existing global health inequities.

Finally, a balance must be struck between the global dissemination of existing diagnostic and treatment technologies and the development of new technologies for global health. Our review revealed how pilot studies of AI in global oncology are particularly common. Although pilot studies can provide an important starting point, if not followed by a robust evaluation to measure the clinical effectiveness of these technologies, which occurs in only a minority of cases [176], these applications will remain an ineffective means of addressing global health disparities in cancer care. Moreover, it has been noted that most cancer deaths occurring in LMICs are due to a lack of access to already present and cost-effective diagnostic and treatment strategies, as opposed to the latest cutting-edge technology [177,178]. Exploratory research into novel technologies in global oncology may detract from the need to develop cost-effective ways to disseminate existing evidence-based technologies in cancer care.

The second major theme noted in our review was the use of biased AI algorithms in clinical decision-making, which may impact the quality and accuracy of decisions and consequently lead to adverse health outcomes for patients [179]. One theme identified in our review was the use of AI algorithms to standardize and reduce bias in clinical decision-making in oncology. One high-profile example is Watson for Oncology, an AI decision support system that has been proposed as a method of standardizing clinical decisions. Watson for Oncology uses natural language processing to provide treatment recommendations in oncology based on the latest scientific literature. Select studies have shown high concordance between treatment plans produced by Watson for Oncology and recommendations from multidisciplinary tumor boards [180-182]. Previous criticisms of this technology have pointed toward problems using concordance to assess the capability of AI technologies, such as Watson for Oncology, because it simply assesses its ability to reproduce specific expert knowledge while not evaluating the validity of applying this knowledge in different contexts [183,184]. Treatment recommendations are based on the current literature as opposed to novel findings produced by the AI system, and preexisting biases found in data sets will be exacerbated rather than mitigated in an automation process. As Murphy et al [185] note, concerns regarding implicit bias becoming embedded in AI algorithms have been widely voiced. The authors noted that implicit biases often reflect preexisting societal values that may exacerbate already-existing health inequities for marginalized populations. Moreover, concerns surrounding lack of transparency in how Watson for Oncology integrates data from heterogeneous sources to arrive at decisions, including the influence of implicit value judgments found in different oncology guidelines, require further attention, specifically focusing on how this might impact the application of Watson for Oncology in different global contexts and its effects on health equity.

Despite the pressing nature of these concerns, the paucity of studies on biased AI algorithms in our search was surprising. Many AI applications identified in our study were trained on selecting data sets from single institutions, creating a high risk of bias, which should be a pressing concern, given that algorithmic bias can exacerbate health inequities [140,186]. A prominent cause of bias is the lack of consideration of the different contexts in which an algorithm is developed and subsequently deployed. Academics weary of these concerns have argued that a generalizable AI model should be developed from data reflecting the diversity of patients on whom it will be applied, yet “most health organizations lack the data infrastructure required to collect the data needed to optimally train these algorithms” [186,187]. Patterns detected when these algorithms are trained on majority groups may result in decreased accuracy when applied to minority groups [188]. For instance, most AI algorithms for diagnosing melanoma are trained on white-skinned individuals and thus may underperform in diagnosing lesions on persons of color [189]. Panch et al [186] note that solutions to these contextual problems involve establishing the appropriate context for which the algorithms will be used. Our literature review identified some proposed solutions, such as the application of transfer learning to improve outcomes for populations with data sparsity; stratification of groups based on race and ethnicity to mitigate bias; and the need for multidisciplinary collaboration between clinicians, engineers, social scientists, and ethicists to aid in the contextual design and development of AI algorithms to mitigate biases [108,135,138,190].

The final theme identified in our review was the use of AI to examine determinants of health outcomes in oncology. Social determinants of health such as education, neighborhood, social community, and socioeconomic status impact health outcomes in oncology [191], and the complex interactions between these variables suggest a potential area for AI applications. Several studies in our review applied AI to analyze large volumes of data to help elucidate the social determinants of cancer outcomes. The identification of social determinants of health can help support more comprehensive strategies to improve health equity in underserved populations [192].

However, as noted by several researchers comparing the use of AI with traditional statistical methods to analyze large amounts of data, it is not always clear what benefits the former provides over the latter to investigate the social determinants of health [145]. A systematic review compared the performance of logistic regression and ML in clinical prediction models and found no evidence that ML performs better than logistic regression [193]. Moreover, traditional statistical models are often easier to interpret than complex, multilayered ML models. Trade-offs between accuracy and transparency have been widely discussed in the literature on AI [194,195] and should be considered when deciding the method of analysis for a given research question. Appropriate and sufficient justification for the use of ML models in clinical and epidemiological oncology research is imperative.

Ethical concerns regarding the use of AI to analyze large amounts of health care data have also been raised in the literature. In establishing a research ethics framework for health care ML, McCraden et al [196,197] note how AI can influence 2 phases of health care research: hypothesis generation and hypothesis testing. AI research focused on hypothesis generation applies computational techniques to large data sets to explore models with potential clinical applicability [197]. This type of exploratory research raises important ethical issues, such as the protection of data privacy and tensions between enabling ready access to data and the requirements of informed consent [197]. Most articles from our review under this theme fit into the hypothesis generation phase and used AI for exploratory research on the determinants of health outcomes in oncology. In our review, the discussion of ethical issues in data privacy versus the need to enable ready access to data was sparse, despite the importance of such considerations in exploratory AI research on social determinants of health, which often requires large amounts of personal health information and other sensitive data. Moreover, as previously emphasized by advocates for equity in AI, exploratory AI research also entails an ethical commitment to ensure representative data sets, including minorities and “data-impoverished” groups, to avoid biased and misleading findings [198]. Few articles from our review addressed these ethical concerns [91,128,199-201].

Finally, it is important to note that the use of AI in health care research lends itself to the analysis of quantitative and categorical data, limiting its ability to understand and explain many social and health-related phenomena. The use of race and other contested social categories in AI algorithms often relies on third-party classification in a way that risks misrepresentation [202]. Therefore, although AI may offer insights into the social determinants of health in oncology, such tools do not obviate the need for other methods, including qualitative methods, in cancer research.

### Limitations

Our study has several limitations. First, the application of AI in oncology is a rapidly evolving field, and as such, the themes and gaps identified in our scoping review are necessarily provisional. To help mitigate this, we conducted a secondary search 9 months after our initial search, which yielded an additional 949 abstracts, of which 21 (2.2%) met the inclusion criteria. Despite this rapid evolution, our findings provide insights into the current state of the literature on the impact of AI on health equity in oncology and may also provide a lens for the early integration of AI technologies in health care more generally. Second, we focused our search strategy in the field of oncology and contemporary cancer research; while the themes and gaps highlighted may be illustrative of more general health equity issues arising from the integration of novel technologies in health care at large, there are likely additional themes pertaining to other areas of health care not covered by our review. Finally, our search was limited to records written in English; we were unable to include articles published in other languages, which may bias our findings toward research conducted in and themes prevalent in the English-speaking world; further work could involve a team of multilingual researchers to shed light on themes from non–English-language research literature.

### Conclusions

In conclusion, we conducted a scoping review to characterize and assess the literature on the impact of AI on health equity in oncology. Our analysis identified 3 general themes related to how AI can be used to address health disparities, how bias might be mitigated or exacerbated by AI algorithms, and how AI can help investigate the social determinants of health. Our review also identified several gaps and areas in need of further research. These include fostering greater collaboration between HICs and LMICs in the design of AI technologies, ensuring representation in training data sets, considering the context of algorithmic development and application to mitigate bias, and recognizing ethical and methodological issues arising from the use of AI to investigate the determinants of cancer outcomes. As AI applications in oncology continue to expand, attention to these issues will be critical to prevent harm and ensure equitable distribution of the potential benefits of these technologies.

## Appendix

Multimedia Appendix 1 Full search strategy.

Multimedia Appendix 2 Theme 1 articles: artificial intelligence to address health disparities.

Multimedia Appendix 3 Theme 2 articles: artificial intelligence and bias.

Multimedia Appendix 4 Theme 3 articles: artificial intelligence and determinants of health.

Multimedia Appendix 5 Multiple-theme articles.

## Abbreviations

AI: artificial intelligence
HIC: high-income country
HPV: human papillomavirus
LMIC: low- and middle-income country
ML: machine learning
PRISMA-ScR: Preferred Reporting Items for Systematic Reviews and Meta-Analyses extension for Scoping Reviews
