# Supplementary material for: The Impact of Artificial Intelligence on Health Equity in Oncology: Scoping Review
Source: J Med Internet Res. 2022 Nov 1;24(11):e39748. doi: 10.2196/39748 (PMC9667381; doi:10.2196/39748)
Supplement: Multimedia Appendix 1 [file jmir_v24i11e39748_app1.docx]

Supplemental Appendix 1: Search Strategy

The Impact of Artificial Intelligence (AI) on Equity in Oncology

**Database: Ovid MEDLINE(R) ALL <1946 to December 03, 2020>**

Search Strategy:

--------------------------------------------------------------------------------

1 exp algorithms/ or algorithm$.mp. or (artificial$ adj3 intelligence$).tw,kf. (485160)

2 ((bat or evolutionary or gravitational$ afj2 search$ or (imperialist$ adj2 competitive$) or firefly or (swarm$ adj2 intelligence$) or memetic$ or heuristic$) adj3 algorithm$).tw,kf. (3244)

3 ((simulated$ adj2 annealing$) or (Tabu$ adj2 search$)).tw. (3413)

4 (metaheuristic$ or (computer$ adj3 heuristic$) or meta-heuristic$ or hyper?heuristic$).tw,kf. (604)

5 ((automated$ adj3 reasoning$) or (ambient$ adj3 intelligence$) or ((multicriteria$ or (multiple$ adj2 criteria$) or multi-criteria$) adj3 decision$) or ((multicriteria$ or (multiple$ adj2 criteria$) or (multiple$ adj3 objective$) or multiobjective$ or multi-objective$ or multi-criteria$) adj3 optimization$)).tw. (2966)

6 ((machine$ adj2 learning$) or (deep$ adj2 learning$) or (computational$ adj3 Intelligence$) or (predictive$ adj3 analytic*)).tw,kf. (48789)

7 ((support$ adj2 vector$) or (convolution$ adj3 neural$ adj3 net$1) or (neural$ adj2 network*)).tw,kf. (70452)

8 (bioinformatics$ or bayesian$).tw,kf. (101830)

9 ((computer$ adj3 vision$) or (visuali$ adj5 (cluster$ or heat?map$))).tw,kf. (5507)

10 or/1-9 (616439)

11 ((resource$ or cost$) adj3 (low$ or limited$ or inadequate$ or poor$)).tw,kf. (135100)

12 10 and 11 (7238)

13 exp Socioeconomic Factors/ or exp Rehabilitation/ or exp insurance/ or exp disability evaluation/ or exp Disabled Persons/ or Absenteeism/ (983391)

14 (socioeconomic$ or (socio$ adj2 economic$)).mp. (248432)

15 (rehabilitation$ or habilitation$ or (activit$ adj3 daily adj3 living$)).mp. (386666)

16 (social$ adj3 (mobil$ or class$)).tw. (13527)

17 ((social$ or living$) adj3 condition$).mp. (26136)

18 (social$ adj3 circumstanc$).tw. (1818)

19 (vocation$1 or occupation$1).tw. (40927)

20 (unemploy$ or employment$ or employe$ or reemployment$ or re-employment$ or worker$).tw. (623993)

21 (income$1 or insurance$ or (salary$ or salaries$)).tw. (213342)

22 ((level$ or factor$) adj5 education$).tw. (66626)

23 (poverty$ or indigen$).tw. (60828)

24 (((family$ or sick$) adj3 leave$) or (health$ adj2 benefit$ adj3 plan$) or (health$ adj5 insurance$) or (work adj3 (absence$ or loss)) or (sick$ adj3 absence$)).mp. (107855)

25 (disabilit$ or disabled$ or disablement or absenteeism$ or compensation$ or economic$ or claims).tw. (574881)

26 "Social Determinants of Health"/ or ((social$ adj5 determinant$) and health$).tw,kf. (11559)

27 or/13-26 (2328796)

28 10 and 27 and (hospital$ or patient$ or health$).mp. [Concept: AI PLUS concept: Socioeconomic Status/ Socioeconomic Inequity ] (24408)

29 Developing Countries/ or ((countries or country$ or nation$1 or societies or society$) adj5 (developing or ((less$1 or least$ or under$) adj3 developed) or middle-income$ or low-income$ or (low$ adj2 middle$ adj3 income$) or third-world$)).tw,kf. (154664)

30 exp american native continental ancestry group/ or alaska natives/ or indians, central american/ or indians, north american/ or indians, south american/ or inuits/ or oceanic ancestry group/ (31854)

31 exp continental population groups/ or exp ethnic groups/ (299207)

32 (First Nations$ or metis$ or Inuit$ or aboriginal$ or indigenous$).mp. (46763)

33 eh.fs. [Ethnology] (164689)

34 or/29-33 (551374)

35 10 and 34 and health$.tw,kf. (1882)

36 or/1-9 (616439)

37 (racial$ or race$1 or ethnic$).tw,kf. (249345)

38 (racial$ or race$1 or ethnic$).tw,kw. and (sex or gender$ or female$ or women$ or ((female$ or women$) and male)).tw. (115106)

39 36 and (or/37-38) (3902)

40 (sex or gender$).tw. and (female$ or women$ or ((female$ or women$) and male)).tw,kw. (339344)

41 (sex or gender$ or female$ or women$ or ((female$ or women$) and male)).ti. or (sex or gender$ or female$ or women$ or ((female$ or women$) and male)).ab. /freq=2 (1195974)

42 36 and (or/40-41) (12459)

43 limit 36 to (female or male) (140808)

44 43 and (sex or (female and male) or gender$).tw. (9858)

45 36 and ((marital$ adj3 status$) or (nation$ adj3 origin$)).tw,kf. (177)

46 39 or 42 or 44 or 45 (20412)

47 (discriminat$ or stereotype$ or discrepan$ or equall$ or equalit$ or inequalit$ or disparit$ or personal or bias$2).mp. (1067107)

48 36 and 46 and 47 [Concept: AI PLUS concept: Racial Bias / Gender Bias ] (2828)

49 Healthcare Disparities/ or Health Status Disparities/ or exp Health Services Accessibility/ (138430)

50 (health$ and (equalit$ or equity$ or inequalit$ or disparit$ or differences)).tw,kf. (348775)

51 or/49-50 (460688)

52 10 and 51 [Concept: AI PLUS concept: Health Equity] (7832)

**53 12 or 28 or 35 or 48 or 52 (39390)**

54 exp Neoplasms/ or (cancer$ or carcinoma$ or neoplas$ or metast$ or tumo?r$ or adeno?carcinoma$ or malignan$).tw,kf. (4448057)

55 (SCC or cSCC or squamous$ or metaplasia$ or fibro?adenoma$ or adenoma$ or craniopharyngioma$ or meningioma$).tw,kf. (270525)

56 or/54-55 (4473536)

57 53 and 56 (4819)

58 limit 57 to english language (4619)

**59 limit 58 to yr="2000 -Current" (4440)**

***************************
