# Supplementary material for: The Impact of Artificial Intelligence on Health Equity in Oncology: Scoping Review
Source: J Med Internet Res. 2022 Nov 1;24(11):e39748. doi: 10.2196/39748 (PMC9667381; doi:10.2196/39748)
Supplement: Multimedia Appendix 2 [file jmir_v24i11e39748_app2.docx]

Multimedia Appendix 2

Theme 1 Articles: AI to Address Health Disparities

| Subtheme | Reference Number | Author | Year | Limitations |
| --- | --- | --- | --- | --- |
| **LMIC Applications** |  |  |  |  |
|  | 31 | Ningrum et al | 2021 | Does not address infrastructural needs to effectively apply the AI technology at scale |
|  | 96 | Asiedu et al | 2019 | Does not address infrastructural needs to effectively apply the AI technology at scale |
|  | 94 | Asiedu et al | 2020 | Does not address infrastructural needs to effectively apply the AI technology at scale |
|  | 84 | Asiedu et al | 2017 | Lack of co-design between HIC and LMIC centers |
|  | 89 | Bae et al | 2020 | Lack of co-design between HIC and LMIC centers |
|  | 93 | Castro et al | 2019 | Does not address infrastructural needs to effectively apply the AI technology at scale |
|  | 24 | Choudhary et al | 2021 | Lack of co-design between HIC and LMIC centers |
|  | 124 | Esteva et al | 2017 | Lack of co-design between HIC and LMIC centers |
|  | 148 | Im et al | 2018 | Lack of co-design between HIC and LMIC centers |
|  | 98 | Kisling et al | 2019 | Further prospective studies needed to assess generalizability of AI technology |
|  | 103 | Min et al | 2018 | Lack of co-design between HIC and LMIC centers |
|  | 95 | Parra et al | 2020 | Lack of co-design between HIC and LMIC centers |
|  | 123 | Quang et al | 2016 | Lack of co-design between HIC and LMIC centers |
|  | 37 | Tan et al | 2021 | Further prospective studies needed to assess generalizability of AI technology |
|  | 32 | Tanriver et al | 2021 | Further prospective studies needed to assess generalizability of AI technology |
|  | 27 | Tunthanathip | 2021 | Further prospective studies needed to assess generalizability of AI technology |
|  | 152 | Uthoff et al | 2019 | Further prospective studies needed to assess generalizability of AI technology |
|  | 118 | Uthoff et al | 2018 | Further prospective studies needed to assess generalizability of AI technology |
|  | 136 | Veronese et al | 2021 | Lack of co-design between HIC and LMIC centers |
|  | 36 | Wadhawan et al | 2011 | Lack of co-design between HIC and LMIC centers |
|  | 88 | Xue et al | 2020 | Lack of co-design between HIC and LMIC centers |
|  | 126 | Briercheck et al | 2019 | Does not address infrastructural needs to effectively apply the AI technology at scale |
|  | 38 | Cerrato et al | 2020 | Does not address infrastructural needs to effectively apply the AI technology at scale |
|  | 85 | Holmström et al | 2021 | Further prospective studies needed to assess generalizability of AI technology |
|  | 97 | Hunt et al | 2021 | Does not address infrastructural needs to effectively apply the AI technology at scale |
|  | 86 | Kudva et al | 2018 | Further prospective studies needed to assess generalizability of AI technology |
|  | 29 | Mohamed et al | 2021 | Further prospective studies needed to assess generalizability of AI technology |
|  | 130 | Valvert et al | 2021 | Further prospective studies needed to assess generalizability of AI technology |
|  | 33 | Warin et al | 2021 | Does not address infrastructural needs to effectively apply the AI technology at scale |
|  | 119 | Song et al | 2021 | Further prospective studies needed to assess generalizability of AI technology |
|  | 125 | Wu et al | 2020 | Further prospective studies needed to assess generalizability of AI technology |
|  | 30 | James et al | 2021 | Further prospective studies needed to assess generalizability of AI technology |
|  | 121 | Adams et al | 2021 | Cost savings of AI technology are likely overestimated given study design |
|  | 117 | Zhang et al | 2020 | Does not address infrastructural needs to effectively apply the AI technology at scale |
|  | 153 | Song et al | 2018 | Further prospective studies needed to assess generalizability of AI technology |
|  | 102 | Love et al | 2018 | Does not address infrastructural needs to effectively apply the AI technology at scale |
|  | 35 | Jin et al | 2020 | Lack of co-design between HIC and LMIC centers |
|  | 28 | Rocha et al | 2020 | Perspectives on AI technology are derived from a small sample of physicians at a single center |
|  | 154 | Ilhan et al | 2021 | Lack of co-design between HIC and LMIC centers |
|  | 90 | Ajenifuja et al | 2020 | AI technology may incur additional financial and logistical strain in LMICs |
|  | 127 | Ilhan et al | 2020 | Limited discussion of the generalizability of AI models outside training datasets |
|  | 149 | Kar et al | 2020 | Limited discussion of the generalizability of AI models outside training datasets |
|  | 122 | Otero et al | 2020 | Further prospective studies needed to assess generalizability of AI technology |
|  | 106 | Lehman et al | 2018 | Limited discussion of the generalizability of AI models outside training datasets |
|  | 129 | Anirvan et al | 2020 | Limited discussion of collaboration between HIC and LMIC centers |
|  | 91 | Xue et al | 2020 | Limited discussion of the infrastructural needs to effectively apply AI technology in LMICs |
|  | 92 | Yang et al | 2021 | Limited discussion of the infrastructural needs to effectively apply AI technology in LMICs |
|  | 34 | Chen et al | 2021 | Lack of co-design between HIC and LMIC centers |
|  | 87 | Hu et al | 2020 | Further prospective studies needed to assess generalizability of AI technology |
|  | 26 | Snuderl et al | 2021 | Limited discussion of the generalizability of AI models outside training datasets |
|  | 104 | Bakre et al | 2018 | Lack of co-design between HIC and LMIC centers |
|  | 131 | Timmerman et al | 2020 | Lack of co-design between HIC and LMIC centers |
|  | 25 | Zhang et al | 2019 | Limited discussion of the generalizability of AI models outside training datasets |
| **HIC Applications** |  |  |  |  |
|  | 120 | Rock et al | 2019 | Further prospective studies needed to assess generalizability of AI technology |
|  | 23 | DeStephano et al | 2020 | Limited discussion of the infrastructural needs to effectively apply AI technology in LMICs |
|  | 105 | Lehman et al | 2021 | Limited discussion of the infrastructural needs to effectively apply AI technology |
| **Both Subthemes** |  |  |  |  |
|  | 83 | Schlemmer et al | 2018 | Limited discussion on collaboration between HIC and LMIC centers |
|  | 128 | Ngwa et al | 2020 | Limited discussion on the generalizability of AI models outside training datasets |
