# Supplementary material for: The Impact of Artificial Intelligence on Health Equity in Oncology: Scoping Review
Source: J Med Internet Res. 2022 Nov 1;24(11):e39748. doi: 10.2196/39748 (PMC9667381; doi:10.2196/39748)
Supplement: Multimedia Appendix 3 [file jmir_v24i11e39748_app3.docx]

Multimedia Appendix 3

Theme 2 Articles: AI and Bias

| Subtheme | Reference Number | Author | Year | Limitation |
| --- | --- | --- | --- | --- |
| **Uncovering Biases** |  |  |  |  |
|  | 134 | Howard et al | 2021 | Limited to analysis of bias in a single database |
|  | 138 | Gao et al | 2020 | Does not address root causes of data inequality between ethnic groups |
|  | 140 | Gilson et al | 2021 | Findings are from a single centre and may lack generalizability |
|  | 39 | Lee et al | 2021 | Limited to analysis of bias in a single database |
|  | 41 | Khoury et al | 2018 | Limited discussion of how to address issues stemming from biased datasets |
|  | 40 | Matin et al | 2021 | Limited discussion of how to address issues stemming from biased datasets |
|  | 44 | Bradley et al | 2019 | Limited discussion of how to address issues stemming from biased datasets |
|  | 42 | Guo et al | 2022 | N/A |
|  | 139 | Khor et al | 2021 | Findings are from a single centre and may lack generalizability |
|  | 79 | Lee et al | 2021 | Findings are from a single centre and may lack generalizability |
| **Mitigating Biases** |  |  |  |  |
|  | 43 | Chen et al | 2019 | Does not examine the impact of AI decision support on clinical outcomes |
|  | 132 | Lee et al | 2022 | N/A |
|  | 135 | Okoji et al | 2021 | N/A |
|  | 107 | Cobb et al | 2021 | Limited discussion of how to address issues stemming from biased datasets |
|  | 82 | Giannitrapani et al | 2020 | Limited discussion on how to address issues stemming from biased datasets |
| **Both Subthemes** |  |  |  |  |
|  | 108 | Thrall et al | 2021 | N/A |
