# Supplementary material for: The Impact of Artificial Intelligence on Health Equity in Oncology: Scoping Review
Source: J Med Internet Res. 2022 Nov 1;24(11):e39748. doi: 10.2196/39748 (PMC9667381; doi:10.2196/39748)
Supplement: Multimedia Appendix 4 [file jmir_v24i11e39748_app4.docx]

Multimedia Appendix 4

Theme 3 Articles: AI and Determinants of Health

| Subtheme | Reference Number | Author | Year | Limitation |
| --- | --- | --- | --- | --- |
| **Social Determinants** |  |  |  |  |
|  | 145 | Muhlestein et al | 2017 | Quantitative focus may benefit from additional qualitative research |
|  | 51 | Mahmood et al | 2020 | Lack of discussion surrounding issues with data privacy |
|  | 76 | Cheng et al | 2013 | Limited justification of the use of AI over conventional statistics |
|  | 58 | Sha et al | 2020 | Dataset lacks variables important for effective AI model prediction |
|  | 57 | Shew et al | 2019 | Dataset lacks variables important for effective AI model prediction |
|  | 72 | Hanson et al | 2019 | Quantitative focus may benefit from additional qualitative research |
|  | 55 | Hassoon et al | 2020 | Lack of discussion surrounding issues with data privacy |
|  | 52 | Hu et al | 2021 | Limited justification of the use of AI over conventional statistics |
|  | 150 | Lynch et al | 2020 | Limited justification of the use of AI over conventional statistics |
|  | 71 | Pino et al | 2019 | Limited discussion of specific variables used to train AI models |
|  | 45 | Pramesh et al | 2020 | Limited justification of the use of AI over conventional statistics |
|  | 69 | Raffenaud et al | 2019 | Limited justification of the use of AI over conventional statistics |
|  | 49 | Sultana et al | 2022 | Limited to analysis of participant perspectives using AI technology |
|  | 73 | Aghdam et al | 2018 | Limited justification of the use of AI over conventional statistics |
|  | 147 | Aghdam et al | 2020 | Limited justification of the use of AI over conventional statistics |
|  | 100 | Asadi et al | 2020 | Did not discuss representative data collection |
|  | 68 | Gajra et al | 2021 | Lack of discussion surrounding issues with data privacy |
|  | 64 | He et al | 2020 | Focus on quantitative data needs to be supplemented by qualitative research |
|  | 109 | Hou et al | 2020 | Focus on quantitative data needs to be supplemented by qualitative research |
|  | 75 | Juacaba et al | 2020 | Focus on quantitative data needs to be supplemented by qualitative research |
|  | 110 | Sidey-Gibbons et al | 2021 | Lack of discussion surrounding issues with data privacy |
|  | 111 | Sidey-Gibbons et al | 2020 | Lack of discussion surrounding issues with data privacy |
|  | 101 | Tossas et al | 2020 | Lack of discussion surrounding issues with data privacy |
|  | 112 | Zhong et al | 2020 | Lack of discussion surrounding issues with data privacy |
|  | 53 | Zhu et al | 2019 | Lack of discussion surrounding issues with data privacy |
|  | 113 | Wheeler et al | 2020 | Lack of discussion surrounding issues with data privacy |
|  | 151 | Manz et al | 2020 | Focus on quantitative data needs to be supplemented by qualitative research |
|  | 48 | Das et al | 2019 | Lack of discussion surrounding issues with data privacy |
|  | 143 | An et al | 2021 | Did not discuss representative data collection |
|  | 66 | Liao et al | 2021 | Did not discuss representative data collection |
|  | 59 | Galadima et al | 2020 | Did not discuss representative data collection |
|  | 144 | Bibault et al | 2020 | Did not discuss representative data collection |
|  | 65 | Benci et al | 2019 | Did not discuss representative data collection |
|  | 146 | Muhlestein et al | 2018 | Focus on quantitative data needs to be supplemented by qualitative research |
|  | 50 | Sim et al | 2020 | Lack of discussion surrounding issues with data privacy |
|  | 114 | Lehman et al | 2021 | Focus on quantitative data needs to be supplemented by qualitative research |
|  | 80 | Agrawal et al | 2021 | Limited justification of the use of AI over conventional statistics |
|  | 81 | Kim et al | 2021 | Limited justification of the use of AI over conventional statistics |
|  | 60 | Cherry et al | 2020 | Focus on quantitative data needs to be supplemented by qualitative research |
|  | 63 | Tran et al | 2019 | Limited justification of the use of AI over conventional statistics |
|  | 70 | Muhammad et al | 2019 | Limited justification of the use of AI over conventional statistics |
|  | 67 | Fiano et al | 2021 | Limited justification of the use of AI over conventional statistics |
|  | 4 | Kann et al | 2021 | Limited discussion of potential adverse impacts of AI on health equity |
| **Genetic Determinants** |  |  |  |  |
|  | 61 | Dillon et al | 2013 | Did not discuss representative data collection |
|  | 141 | Li et al | 2020 | Focus on quantitative data needs to be supplemented by qualitative research |
|  | 142 | van Dams et al | 2019 | Focus on quantitative data needs to be supplemented by qualitative research |
|  | 46 | Chen et al | 2021 | Focus on quantitative data needs to be supplemented by qualitative research |
|  | 62 | Ding et al | 2014 | Focus on quantitative data needs to be supplemented by qualitative research |
|  | 47 | Pino et al | 2021 | Lack of discussion surrounding issues with data privacy |
|  | 74 | Ramakrishnan et al | 2018 | Focus on quantitative data needs to be supplemented by qualitative research |
|  | 115 | Yang et al | 2020 | Focus on quantitative data needs to be supplemented by qualitative research |
|  | 56 | Kaplan et al | 2020 | Limited justification of the use of AI over conventional statistics |
| **Both Subthemes** |  |  |  |  |
|  | 99 | Azarianpour et al | 2021 | Did not discuss representative data collection |
|  | 133 | D' Souza et al | 2010 | Lack of discussion surrounding issues with data privacy |
|  | 54 | Cirstea et al | 2019 | Lack of discussion surrounding issues with data privacy |
