# Supplementary material for: The Impact of Artificial Intelligence on Health Equity in Oncology: Scoping Review
Source: J Med Internet Res. 2022 Nov 1;24(11):e39748. doi: 10.2196/39748 (PMC9667381; doi:10.2196/39748)
Supplement: Multimedia Appendix 5 [file jmir_v24i11e39748_app5.docx]

Multimedia Appendix 5

Articles touching on multiple themes.

| Themes Covered | Reference Number | Author | Year | Limitation |
| --- | --- | --- | --- | --- |
| 2, 3 | 78 | Greatbatch et al. | 2019 | N/A - Review |
| 1, 2, 3 | 116 | Mema et al. | 2020 | N/A - Review |
| 1, 2, 3 | 137 | Pangti et al. | 2021 | AI model does not incorporate patient medical history into its image analysis |
| 1, 2, 3 | 77 | Urman et al. | 2018 | Limited discussion of potential adverse impacts of AI on health equity |
